# Understanding Pornography Addiction in India: Insights from a Retrospective, Observational Study

**DOI:** 10.1192/bjo.2025.10153

**Published:** 2025-06-20

**Authors:** Neeharika Jaiswal, Sandip Deshpande, Nikunj Gokani, Ashima Sahore, Tanha Gowande

**Affiliations:** 1 Allo Health, Noida, India; 2 Allo Health, Bangalore, India; 3 Allo Health, Mumbai, India; 4 University of Mumbai, Mumbai, India

## Abstract

**Aims:** This study examines the demographic factors and severity of pornography addiction in India, analysing the impact of age, relationship status, co-morbid conditions, and socio-cultural norms on addiction patterns and treatment outcomes.

**Methods:** A retrospective, observational, cross-sectional study was conducted with 589 participants (583 males, 6 females) aged 18 and older, diagnosed with pornography addiction as per the International Classification of Diseases, 11th revision (Compulsive Sexual Behavior Disorder criteria). Participants sought treatment through Allo Health’s online and offline platforms. Data on demographics, clinical diagnoses, and treatment outcomes were extracted from electronic health records and analysed using IBM Statistical Product and Service Solutions (SPSS-14). Treatment options included pharmacological interventions (Selective Serotonin Reuptake Inhibitors, bupropion) and psychotherapy (cognitive-behavioural therapy, motivational interviewing, relapse prevention strategies).

**Results:** The majority (374) were under 30, with a mean age of 28.98 years. Most were single (287), followed by married (167), in relationships (112), and divorced (6). Regarding sexual orientation, 568 identified as heterosexual, 15 as bisexual, 2 as homosexual, and 4 preferred not to disclose.

A strong association was found between younger age and higher addiction severity (p=0.004), suggesting early exposure to explicit content and digital overuse contribute to compulsive consumption. Single individuals exhibited significantly higher addiction severity than those in relationships or married (p<0.001), likely due to limited sexual expression opportunities.

Treatment varied, with 338 receiving medication alone, 207 undergoing therapy only, and 42 receiving a combination. Younger individuals were more likely to receive therapy, reflecting growing acceptance of psychological treatment. Two individuals were ineligible for treatment.

**Conclusion:** This study highlights the complexity of pornography addiction in India, showing a strong link between age, relationship status, and addiction severity. Younger individuals are particularly vulnerable due to early exposure, poor coping mechanisms, and excessive digital engagement. Socio-cultural factors influence addiction, with single individuals facing limited sexual expression in a conservative society. Stigma surrounding sexual health discourages help-seeking, especially in collectivist cultures.

The findings emphasize the need for culturally sensitive interventions, including awareness campaigns, digital literacy programmes, and public health initiatives. Future research should focus on longitudinal studies, gender disparities, and the impact of emerging digital technologies on addiction trends.

